# Impact of Maternal Thyroperoxidase Status on Fetal Body and Brain Size

**DOI:** 10.1155/2014/872410

**Published:** 2014-01-29

**Authors:** Roneé E. Wilson, Hamisu M. Salihu, Maureen W. Groer, Getachew Dagne, Kathleen O'Rourke, Alfred K. Mbah

**Affiliations:** ^1^Department of Epidemiology and Biostatistics, College of Public Health, University of South Florida, 13201 Bruce B. Downs, MDC56, Tampa, FL 33612, USA; ^2^Division of Maternal Fetal Medicine, Department of Obstetrics and Gynecology, College of Medicine, University of South Florida, 2 Tampa General Circle, STC, 6th Floor Tampa, FL 33606, USA; ^3^College of Nursing, University of South Florida, 12901 Bruce B. Downs, MDC22, Tampa, FL 33612, USA

## Abstract

The obstetric consequences of abnormal thyroid function during pregnancy have been established. Less understood is the influence of maternal thyroid autoantibodies on infant outcomes. The objective of this study was to examine the influence of maternal thyroperoxidase (TPO) status on fetal/infant brain and body growth. Six-hundred thirty-one (631) euthyroid pregnant women were recruited from prenatal clinics in Tampa Bay, Florida, and the surrounding area between November 2007 and December 2010. TPO status was determined during pregnancy and fetal/infant brain and body growth variables were assessed at delivery. Regression analysis revealed maternal that TPO positivity was significantly associated with smaller head circumference, reduced brain weight, and lower brain-to-body ratio among infants born to TPO+ white, non-Hispanic mothers only, distinguishing race/ethnicity as an effect modifier in the relationship. No significant differences were noted in body growth measurements among infants born to TPO positive mothers of any racial/ethnic group. Currently, TPO antibody status is not assessed as part of the standard prenatal care laboratory work-up, but findings from this study suggest that fetal brain growth may be impaired by TPO positivity among certain populations; therefore autoantibody screening among high-risk subgroups may be useful for clinicians to determine whether prenatal thyroid treatment is warranted.

## 1. Introduction

Thyroid dysfunction is one of the most common endocrine disorders in women of childbearing age [1], second only to diabetes mellitus. Approximately 2-3% of women are diagnosed prenatally with abnormal thyroid function; however, a greater number may go undetected due to lack of consensus on testing and treatment modalities during pregnancy [2–4]. Normal maternal thyroid function is critical for early fetal development, as the fetus does not produce thyroid hormones until the end of the first trimester (~12–14 weeks gestation) and, prior to that time, is solely dependent on the mother's hormone supply [5–7]. The impact of thyroid dysfunction, particularly hypothyroidism, during pregnancy is well documented [8, 9], and the associated adverse fetal/infant outcomes range from preterm delivery to fetal death [10–14]. Abnormal maternal thyroid hormone levels during gestation are also linked to long-term effects in older offspring including delayed learning, lowered IQ, and hearing deficits [14–17].

A number of women may be biochemically euthyroid or exhibit thyroid hormone levels within normal limits but test positive for thyroid autoantibodies such as thyroperoxidase (TPO) antibody. In fact, it is estimated that 10% of pregnant women are TPO positive [15]; however, fewer studies have assessed the influence of TPO status on fetal/infant outcomes among euthyroid mothers. Limited research tends to suggest that TPO positivity, independent of abnormal thyroid levels, may increase the risk of placental abruption, spontaneous miscarriage, and perinatal death [11, 13, 18–24]. Even fewer studies have assessed the impact of maternal TPO antibody status on infant specific variables such anthropometric measurements at delivery although these studies have produced conflicting results [21, 25]. To further explore the relationship between maternal autoantibody status and infant outcomes, this study uniquely examined the influence of maternal TPO status on fetal/infant brain growth at delivery, which has been linked to cognitive function in childhood [26, 27]. This project was undertaken with the following hypotheses: (1) at delivery, newborns of TPO+ mothers will exhibit impaired body growth as indicated by reduced birth weight and birth length; (2) at delivery, infants born to TPO+ mothers will exhibit impaired brain growth as exhibited by reduced head circumference and calculated brain weight.

## 2. Methods

### 2.1. Participants

Pregnant women (*N* = 631) were recruited from prenatal clinics in Tampa, Florida, and the surrounding area between November 2007 and December 2010. Women were eligible for participation in the study if they were between 18 and 45 years of age, 16 to 25 weeks gestation, able to understand and speak the recruiter's language (English or Spanish), and essentially healthy without plans to terminate the pregnancy or relocate prior to 6 months postpartum. Exclusion criteria included known autoimmune disease, previous thyroid disease, presence of chronic diseases/conditions including HIV, use of medications that affect immunity, mental illness, body mass index (BMI) <20, current multiple gestation, current pregnancy product of invitro fertilization (IVF), and fetal abnormalities. All women were biochemically euthyroid. Thyroperoxidase antibody status was measured for all participants at the time of enrollment and women were classified as TPO positive or negative. Thyroid stimulating hormone (TSH) levels were measured for all TPO positive women at the time of enrollment. The study was approved by the University of South Florida Institutional Review Board. All participants gave full written informed consent.

### 2.2. Exposure Assessment

Thyroperoxidase antibody (TPO) status was the exposure of interest. TPO status was determined in 631 plasma samples according to kit directions by ELISA (ORGENTEC, Mainz, Germany) using standards and controls. All samples were collected in duplicate and titers recorded. The coefficient of variation was always less than 5%. TPO antibody titer greater than 20 IUs/mL was used as the cutoff value for determining positivity, since a value from 0 to 20 is considered within normal range [13]. Based on TPO titers, women were categorized as TPO positive or TPO negative. The mean TPO titers for each group were 75.6 ± 59.2 and 9.4 ± 4.5, respectively.

### 2.3. Outcome Assessment

Newborn anthropometric measurements were retrieved from maternal delivery records including ultrasound-derived gestational age in weeks, birth weight (grams), birth length (centimeters (cm)), head circumference (cm), abdominal circumference (cm), and chest circumference (cm). Infant head circumference at birth was used to derive two additional indices of fetal brain size: brain weight, and brain-to-body ratio (BBR). Brain weight was estimated from the following formula: brain weight (g) = 0.037 × head circumference (cm)^2.57^, which is derived from the National Institute of Neurological and Communicative Disorders and Stroke's Collaborative Perinatal Project [28]. Brain-to-body ratio (BBR) was defined as 100 × the ratio of the infant's estimated brain weight to its birth weight. This is the percentage of the infant's birth weight that is estimated to reside in the brain. Since the brain weight is a function of the head circumference, the formula could thus be rewritten in terms of birth weight and head circumference as BBR = 100 × [0.037 × head circumference (cm)^2.57^]/birth weight (g). A high BBR is indicative of a higher proportion of birth weight residing in the brain, while a lower BBR indicates a lower percentage of birth weight residing in the brain [29]. Typical values for healthy infants are 9-10% [29].

### 2.4. Study Sample

**Figure 1:**
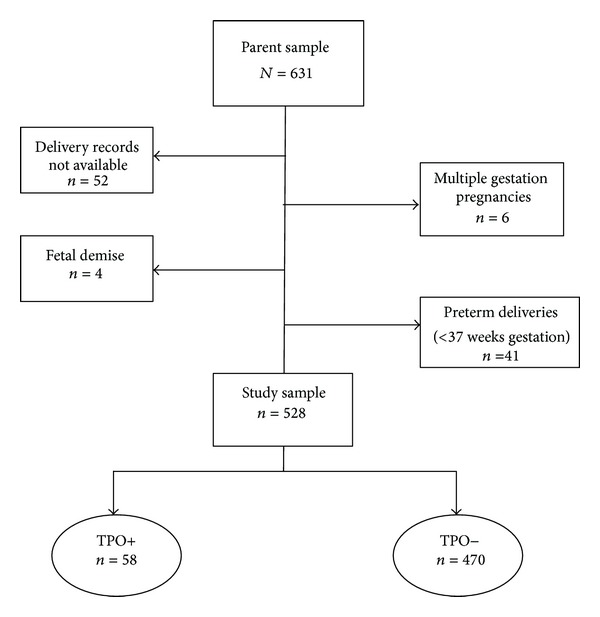
Diagram of study population.

Delivery records for 52 participants were not available at the time of analysis. For the current study, multiple gestation pregnancies (*n* = 6) and pregnancies resulting in fetal demise (*n* = 4) were excluded. To promote homogeneity of the sample and reduce confounding factors, analysis was restricted to term infants only (≥37 weeks gestation), resulting in a final study sample of 528 women who delivered term infants.  Figure 1 provides an overview of the study sample.

### 2.5. Statistical Analysis

Maternal thyroid status was a categorical determinant in this analysis. Chi-square test and two-sample *t*-test were used to assess differences in sociodemographic characteristics between TPO+ and TPO− mothers. Mean differences in growth parameters were examined by maternal TPO status using *t*-test. Multiple regression analysis was used to demonstrate the influence of TPO status on continuous outcomes such as birth weight, birth length, and head circumference. The covariates in the regression models were selected a priori based on information in the published literature. These variables included maternal age, parity, race/ethnicity (White Non-Hispanic; black non-Hispanic, Hispanic and other), marital status, prenatal smoking habits, prepregnancy body mass index (BMI), delivery type and gender of the infant. Several variables were dichotomized in the regression models: parity (nulliparous* or multiparous), marital status (married* or unmarried), smoking habits (smoker or nonsmoker*), pre-pregnancy BMI (overweight (BMI > 25) or nonoverweight*), delivery type (vaginal* or cesarean), and infant gender (male* or female). The  ∗  denotes the referent category for each variable. Additionally, adjusted estimates were derived in all cases by using TPO negative participants as the referent category.

A combination of graphic methods and statistical tests were used to check for violations of the regression assumptions. After fitting the linear regression models to the data, the normality assumption was assessed by visual inspection of the residual normal QQ plots and by use of the Shapiro-Wilk test. Visual inspection of residual scatter plots of outcome variables and errors of prediction were evaluated to ensure that the homoscedasticity assumption was not violated. Variance inflation and tolerance values were used to assess multicollinearity. Data for all outcome variables in the study sample were 99% complete. SAS version 9.3 (SAS Institute, Cary, NC) was used to perform all analyses.

## 3. Results

The final study sample (*n* = 528) comprised pregnant women with a mean age of 28.03 ± 5.85 years (range 18–45). Nearly 48% (*n* = 253) of the sample were white, 59% (*n* = 309) were married, and less than 6% (*n* = 30) were smokers. The mean gestational age at delivery of the term infants retained in the sample was 39.02 ± 1.10 (range 37–41 weeks). Approximately 11% (*n* = 58) of the final sample tested positive for the thyroperoxidase antibody during pregnancy with a mean TSH level of 1.46 ± 1.12.  Table 1 depicts selected sociodemographic characteristics by maternal TPO status. Women who tested positive for the TPO antibody did not differ significantly from their negative counterparts in terms of racial/ethnic background, marital status, smoking habits, or body mass index. TPO+ mothers tended to be older in age and were more likely to deliver female infants; however, neither of these factors achieved statistical significance. Analyses of mean gestational age at delivery showed that white non-Hispanic and Hispanic mothers were more likely to deliver infants at 39+ weeks gestation compared to black mothers and mothers in the “other” racial/ethnic category (data not shown). No differences were noted in delivery type (vaginal versus cesarean) or 1-minute Apgar score. Also, no differences in mean newborn birth weight, birth length, abdominal circumference, or chest circumference were observed between the two groups (Table 2). Nonetheless, small but significant differences were noted for the mean head circumference measurements among infants born to TPO+ mothers versus TPO− mothers (34.45 cm ± 1.34 SD versus 34.86 cm ± 1.45, resp. (*P* = 0.04)). Additionally, infants born to TPO+ mothers had significantly smaller brain weight than those born to TPO− mothers.

**Figure 2:**
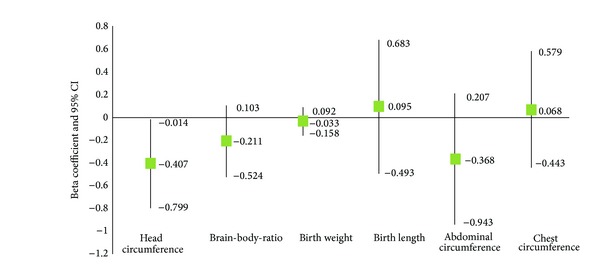
Unadjusted analysis of newborn brain and body growth variables at delivery (brain weight omitted from graph due to scaling differences (*β* = −10.307 (95% CI −20.130–−0.483))) (linear regression beta coefficient and 95% confidence interval (CI)), Tampa Bay, Florida, 2007–2010.

Unadjusted regression analysis for brain growth variables at delivery indicated that infants born to mothers who were TPO+ had a smaller head circumference and reduced brain weight (*β* = −407; standard error (SE) = 0.200; *P* < 0.05 and *β* = −10.307;  SE = 5.001; *P* < 0.05, resp.). Infants born to mothers with TPO positivity also showed a tendency for lower brain-to-body ratio; however, the results were not significant (Figure 2). Unadjusted analysis did not signify an association between TPO status and newborn growth variables (birth weight, birth length, abdominal circumference, or chest circumference).

Table 3 summarizes the adjusted multiple regression results for fetal/infant brain growth variables. After adjusting for several maternal and pregnancy factors including maternal age, smoking habits, and infant weight at birth, the relationship between TPO status and infant head circumference at birth was not significant in the overall population but was highly significant among infants born to white non-Hispanic mothers (*β* = −0.727; standard error (SE) = 0.214; *P* < 0.001) and among those in the “other” racial/ethnic group (*β* = −1.636;  SE = 0.713; *P* < 0.05), distinguishing race/ethnicity as an effect modifier in the relationship between maternal TPO status and fetal brain growth. The association between TPO positivity and reduced fetal/infant brain weight persisted among infants whose mothers were categorized in either the white non-Hispanic or Other racial/ethnic groups. Table 3 also reflects that the association between maternal TPO status and brain-to-body ratio was significant among infants born to TPO+ non-Hispanic white mothers only. In this study population, birth weight, birth length, abdominal circumference, and chest circumference were not associated with TPO status (Table 4).

## 4. Discussion

This study examined the relationship between maternal thyroid peroxidase antibody status and newborn brain and body growth measurements within a cohort of euthyroid pregnant women. The findings indicate that maternal race/ethnicity modifies the relationship between TPO positivity and reduced brain growth measurements at delivery (head circumference, brain weight and brain-to-body ratio). Upon closer examination, the relationship appears to be the most noticeable among white non-Hispanic mothers. Although this analysis indicates that TPO positivity may result in impaired brain growth among infants of mothers in the “other” racial/ethnic category, this finding should be interpreted with caution due to the small sample size of this subgroup (*n* = 37). However, it can be theorized that the differences in brain growth measurements are more marked among white non-Hispanic infants because they are more likely than their nonwhite counterparts to deliver later term (39+ weeks gestation), thus allowing the differences in brain growth variables to be more apparent [30, 31]. However, nonwhite mothers have a tendency to deliver earlier term infants who may not have had the opportunity to reach full potential, thereby masking the effects of TPO influence.

The present analysis did not indicate a significant relationship between TPO status and infant birth weight in this study sample. This finding is contradictory to those studies that have reported increased likelihood of low birth weight infants born to TPO+ mothers [21] and an increased likelihood of large-for-gestational age infants born to TPO+ women [21]. Similar to previously published studies [21], this analysis did not find a difference in the birth length, abdominal circumference, or chest circumference of infants born to TPO+ mothers compared to those born to TPO− mothers.

Although measurement of thyroid antibodies does not give any indication of thyroid function, the presence of TPO antibodies may be associated with decreased thyroid functional reserve during pregnancy [32, 33]. Reduced functional thyroid reserve in combination with the normal physiological changes in pregnancy could contribute to minor alterations in circulating thyroid hormone concentrations while remaining within the normal reference range [34]. Some researchers hypothesize that the presence of TPO antibodies during a time of increasing thyroid hormone demand such as pregnancy implies that the mother may become hypothyroid during gestation and that transient maternal hypothyroidism may ultimately be responsible for the adverse outcomes [33, 35, 36]. However, previous studies have reported the detrimental effects of TPO antibodies independent of abnormal thyroid hormone levels or disorders [13, 21]. Results of this study support this assumption, as our findings indicate that TPO antibodies are associated with reduced brain growth measurements among infants born to a vulnerable subgroup of euthyroid women.

A notable strength of this study is the prospective design, as many of the previous studies were retrospective in nature [10, 35, 37]. Additionally, laboratory analysis of thyroid stimulating hormone (TSH) was used to confirm euthyroid state among participating mothers. The outcome variables were extracted from maternal delivery records; therefore, measurements were not influenced by knowledge of maternal TPO status. One limitation of this study is the lack of laboratory confirmed prenatal TSH data on women in the TPO negative group. This group was presumed to be euthyroid based on the absence of thyroid dysfunction symptoms and the stringency of the study exclusion criteria (e.g., no history of thyroid disease or autoimmune conditions). However, it is unlikely that this limitation influenced the study findings, as thyroid dysfunction in the TPO− group would have biased the results toward the null and resulted in nonsignificant findings in the association between TPO positivity and fetal/infant brain growth. Additionally, postpartum TSH levels were measured on a subsample of the participants resulting in mean values of 3.17 ± 4.73 for TPO+ women (*n* = 47) and 2.05 ± 1.80 for TPO− women (*n* = 41), thus providing additional evidence that the study sample was indeed euthyroid perinatally and hormone levels were within normal limits.

After controlling for several maternal and pregnancy factors, TPO positivity was associated with smaller head circumference, reduced brain weight and lower brain-to-body ratio. However, the influence of TPO status on brain growth was modified by maternal race. Future studies should focus on the identification of genetic variants or single nucleotide polymorphisms (SNPs), which could elucidate the interaction between maternal TPO status and race/ethnicity. Epigenetic analysis may prompt ethnic-based screening for TPO autoantibodies. This is potentially important because findings from recent studies [38] indicate that substitutive treatment with levothyroxine may lower the chance of adverse obstetric outcomes (miscarriage and premature delivery) among euthyroid pregnant women who are positive for TPO autoantibodies.

Considering the long-term implications of impaired fetal/newborn growth, it is important to identify avenues for early prevention and intervention. Maternal thyroid antibody status during pregnancy may be one of those factors that play a role in fetal growth impairment but is currently being overlooked due to lack of consensus on maternal testing and treatment. At present, TPO antibody status is not assessed as part of the standard prenatal care laboratory work-up, but this study suggests that fetal brain growth may be impaired with TPO positivity among certain populations; therefore, autoantibody screening among high-risk subgroups may be useful for clinicians to determine whether prenatal thyroid treatment is warranted.

## Figures and Tables

**Table 1:** Selected sociodemographic characteristics by TPO status of pregnant women in the study.

	TPO positive (*N* = 58)	TPO negative (*N* = 470)	*P* value
Maternal age			
Mean (±SD)	29.31 (±6.28)	28.18 (±5.79)	0.19
Race/ethnicity			
White	29 (50.00%)	224 (47.66%)	0.48
Black	8 (13.79%)	97 (20.64%)	
Hispanic	18 (31.03%)	115 (24.46%)	
Other	3 (5.17%)	34 (7.23%)	
Married			
Yes	37 (63.79%)	272 (58.00%)	0.40
Parity			
Nulliparous	17 (29.82%)	148 (31.56%)	0.79
Smoking			
Yes	2 (3.45%)	28 (6.00%)	0.76
Body mass index (BMI)			
Overweight/obese	39 (67.24%)	301 (64.04%)	0.63

**Table 2:** Selected infant characteristics at delivery by maternal TPO status.

	TPO positive (*N* = 58)	TPO negative (*N* = 470)	*P* value
Gestational age at delivery			
Mean (±SD)	38.98 ± 1.02	39.04 ± 1.11	0.71
Delivery type			
Vaginal	38 (65.52%)	321 (68.30%)	0.67
Gender			
Male	24 (41.38%)	241 (51.28%)	0.15
1-minute Apgar			
Apgar < 7	3 (5.17%)	37 (7.87%)	0.60
Birth weight (gm)			
Mean (±SD)	3374.2 (±418.8)	3407.2 (±461.2)	0.60
Birth Length (cm)			
Mean (±SD)	51.11 (±1.96)	51.02 (±2.17)	0.75
Head circumference (cm)			
Mean (±SD)	34.45 (±1.34)	34.86 (±1.45)	0.04
Brain weight			
Mean (±SD)	331.1 (±33.03)	341.5 (±36.27)	0.04
Brain-to-body ratio			
Mean (±SD)	9.91 (±1.21)	10.12 (±1.14)	0.19
Abdominal circumference (cm)			
Mean (±SD)	30.98 (±2.03)	31.35 (±2.09)	0.21
Chest circumference (cm)			
Mean (±SD)	33.24 (1.92)	33.15 (1.95)	0.74
High birth weight (HBW)	1 (1.72%)	23 (4.89)	0.50
Low birth weight (LBW)	0 (0.00%)	8 (1.70%)	0.61
Small-for-gestational age (SGA)	4 (6.90%)	31 (6.60)	1.00
Large-for-gestational age (LGA)	7 (12.07%)	51 (10.85%)	0.78

**Table 3:** Multiple regression results for the association between maternal TPO status and fetal/infant brain growth variables by race/ethnicity.

	Overall *β* (SE)	White *β* (SE)	Black *β* (SE)	Hispanic *β* (SE)	Other *β* (SE)
Head circumference^Ψ^	−0.313 (0.161)	−0.727*** (0.214)	−0.237 (0.544)	0.471 (0.268)	−1.636** (0.713)
Brain weight^Ψ^	−7.789 (4.0291)	−18.409*** (5.463)	−5.059 (13.346)	11.602 (6.648)	−41.491** (18.001)
Brain-to-body ratio^†^	−0.147 (0.158)	−0.533** (0.220)	0.238 (0.495)	0.333 (0.279)	−1.201 (0.616)

^Ψ^Adjusted for maternal race/ethnicity, smoking habits, maternal age, marital status, parity, maternal obesity, delivery type, infant gender, infant birth weight, and gestational age at delivery.

^†^ Adjusted for maternal race/ethnicity, smoking habits, maternal age, marital status, parity, maternal obesity, delivery type, infant gender, and gestational age.

** and ***indicate significance at 0.05 and 0.01 level, respectively.

**Table 4:** Regression results for fetal/infant body growth variables at delivery.

	Birth weight^†^ *β* (std. error)	Birth length^‡^ *β* (std. error)	Abdominal circumference^‡^ *β* (std. error)	Chest circumference^‡^ *β* (std. error)
TPO status	−0.059 (0.045)	0.205 (0.213)	−0.380 (0.210)	0.149 (0.166)

*R* ^2^	0.5243	0.5198	0.5146	0.6104
Number of Observations	522	522	516	518

^†^Adjusted for maternal race/ethnicity, maternal age, marital status, prenatal smoking habits, parity, prepregnancy body mass index, type of delivery, infant gender, and infant length at delivery.

^‡^Adjusted for maternal race/ethnicity, maternal age, marital status, prenatal smoking habits, parity, prepregnancy body mass index, type of delivery, infant gender, infant birth weight, and gestational age at delivery.

## References

[1] Nader S, Creasy R, Resnik R, Iams J (2004). Thyroid disease and pregnancy. *Maternal-Fetal Medicine*.

[2] Negro R, Schwartz A, Gismondi R, Tinelli A, Mangieri T, Stagnaro-Green A (2010). Universal screening versus case finding for detection and treatment of thyroid hormonal dysfunction during pregnancy. *Journal of Clinical Endocrinology and Metabolism*.

[3] Stagnaro-Green A, Schwartz A (2008). Is universal screening for thyroid disease in pregnancy a cost-effective strategy?. *Nature Clinical Practice Endocrinology and Metabolism*.

[4] Stagnaro-Green A, Abalovich M, Alexander E (2011). Guidelines of the American Thyroid Association for the diagnosis and management of thyroid disease during pregnancy and postpartum. *Thyroid*.

[5] Galofre JC, Davies TF (2009). Autoimmune thyroid disease in pregnancy: a review. *Journal of Women’s Health*.

[6] Forehan S (2012). Thyroid disease in the perinatal period. *Australian Family Physician*.

[7] de Escobar GM, Obregón MJ, del Rey FE (2004). Maternal thyroid hormones early in pregnancy and fetal brain development. *Best Practice & Research Clinical Endocrinology & Metabolism*.

[8] Allan WC, Haddow JE, Palomaki GE (2000). Maternal thyroid deficiency and pregnancy complications: implications for population screening. *Journal of Medical Screening*.

[9] Haddow JE, Palomaki GE, Allan WC (1999). Maternal thyroid deficiency during pregnancy and subsequent neuropsychological development of the child. *New England Journal of Medicine*.

[10] Glinoer D, Soto MF, Bourdoux P (1991). Pregnancy in patients with mild thyroid abnormalities: maternal and neonatal repercussions. *Journal of Clinical Endocrinology and Metabolism*.

[11] Abramson J, Stagnaro-Green A (2001). Thyroid antibodies and fetal loss: an evolving story. *Thyroid*.

[12] Lazarus JH (2005). Thyroid disorders associated with pregnancy: etiology, diagnosis, and management. *Treatments in Endocrinology*.

[13] Prummel MF, Wiersinga WM (2005). Thyroid peroxidase autoantibodies in euthyroid subjects. *Best Practice and Research*.

[14] Wasserman EE, Nelson K, Rose NR (2008). Maternal thyroid autoantibodies during the third trimester and hearing deficits in children: an epidemiologic assessment. *American Journal of Epidemiology*.

[15] Dallas JS (2003). Autoimmune thyroid disease and pregnancy: elevance for the child. *Autoimmunity*.

[16] Lazarus JH (1999). Thyroid hormone and intellectual development: a clinician’s view. *Thyroid*.

[17] Pop VJ, De Vries E, Van Baar AL (1995). Maternal thyroid peroxidase antibodies during pregnancy: a marker of impaired child development?. *Journal of Clinical Endocrinology and Metabolism*.

[18] Negro R, Stagnaro-Green A (2011). Thyroid autoantibodies, preterm birth, and miscarriage. *BMJ*.

[19] Stagnaro-Green A, Glinoer D (2004). Thyroid autoimmunity and the risk of miscarriage. *Best Practice and Research*.

[20] Stagnaro-Green A (2011). Thyroid antibodies and miscarriage: where are we at a generation later?. *Journal of Thyroid Research*.

[21] Männistö T, Vääräsmäki M, Pouta A (2009). Perinatal outcome of children born to mothers with thyroid dysfunction or antibodies: a prospective population-based cohort study. *Journal of Clinical Endocrinology and Metabolism*.

[22] Männistö T, Vääräsmäki M, Pouta A (2010). Thyroid dysfunction and autoantibodies during pregnancy as predictive factors of pregnancy complications and maternal morbidity in later life. *Journal of Clinical Endocrinology and Metabolism*.

[23] Männistö T, Vääräsmäki M, Suvanto E (2011). Pregnancy outcomes in women with thyroid peroxidase antibodies. *Obstetrics and Gynecology*.

[24] Abbassi-Ghanavati M, Casey BM, Spong CY, McIntire DD, Halvorson LM, Cunningham FG (2010). Pregnancy outcomes in women with thyroid peroxidase antibodies. *Obstetrics and Gynecology*.

[25] Bech K, Hertel J, Rasmussen NG (1991). Effect of maternal thyroid autoantibodies and post-partum thyroiditis on the fetus and neonate. *Acta Endocrinologica*.

[26] Gale CR, O’Callaghan FJ, Bredow M, Martyn CN (2006). The influence of head growth in fetal life, infancy, and childhood on intelligence at the ages of 4 and 8 years. *Pediatrics*.

[27] Gross SJ, Kosmetatos N, Grimes CT, Williams ML (1978). Newborn head size and neurological status. Predictors of growth and development of low birth weight infants. *American Journal of Diseases of Children*.

[28] Gilles FH, Leviton A, Dooling EC (1983). *The Developing Human Brain: Growth and Epidemiologic Neuropathology*.

[29] Lindley AA, Gray RH, Herman AA, Becker S (2000). Maternal cigarette smoking during pregnancy and infant ponderal index at birth in the Swedish Medical Birth Register, 1991-1992. *American Journal of Public Health*.

[30] Papiernik E, Alexander GR, Paneth N (1990). Racial differences in pregnancy duration and its implications for perinatal care. *Medical Hypotheses*.

[31] Patel RR, Steer P, Doyle P, Little MP, Elliott P (2004). Does gestation vary by ethnic group? A London-based study of over 122 000 pregnancies with spontaneous onset of labour. *International Journal of Epidemiology*.

[32] Brent GA (2010). *SpringerLink. Thyroid Function Testing*.

[33] Thangaratinam S, Tan A, Knox E, Kilby MD, Franklyn J, Coomarasamy A (2011). Association between thyroid autoantibodies and miscarriage and preterm birth: meta-analysis of evidence. *BMJ*.

[34] Glinoer D (2006). Miscarriage in women with positive anti-TPO antibodies: is thyroxine the answer?. *Journal of Clinical Endocrinology and Metabolism*.

[35] Alexander EK (2011). Autoimmunity: thyroid autoantibodies and pregnancy risk. *Nature Reviews Endocrinology*.

[36] Poppe K, Glinoer D (2003). Thyroid autoimmunity and hypothyroidism before and during pregnancy. *Human Reproduction Update*.

[37] Iijima T, Tada H, Hidaka Y, Mitsuda N, Murata Y, Amino N (1997). Effects of autoantibodies on the course of pregnancy and fetal growth. *Obstetrics and Gynecology*.

[38] Negro R, Formoso G, Mangieri T, Pezzarossa A, Dazzi D, Hassan H (2006). Levothyroxine treatment in euthyroid pregnant women with autoimmune thyroid disease: effects on obstetrical complications. *Journal of Clinical Endocrinology and Metabolism*.

